# Impact of Mediterranean Diet Adherence in Early Pregnancy on Nausea, Vomiting, and Constipation

**DOI:** 10.1007/s10995-025-04078-7

**Published:** 2025-04-07

**Authors:** Isabel Barroso-Ruiz, Naomi Cano-Ibáñez, Rebeca Benito-Villena, Sandra Martín-Peláez, Carmen Amezcua-Prieto

**Affiliations:** 1 Department of Preventive Medicine and Public Health, Faculty of Medicine, University of Granada, Avda. De la Investigación, 11 18016, Granada, Spain; 2 Consortium for Biomedical Research in Epidemiology and Public Health (CIBERESP), Granada, Spain; 3 Institute for Biosanitary Research ibs. GRANADA, Granada, Spain; 4 Obstetrics and Gynecology Service, Hospital Materno-Infantil del Hospital Universitario Virgen de las Nieves. Granada, Granada, Spain; 5 PhD Program in Clinical Medicine and Public Health, International School for Posgraduate Studies, University of Granada, Granada, Spain

**Keywords:** Pregnancy, Nausea, Vomiting, Constipation, Mediterranean diet

## Abstract

**Objectives:**

Common maternal digestive symptoms, such as nausea, vomiting, and constipation during pregnancy, can impair pregnant women’s quality of life. The Mediterranean diet (MedDiet), characterized by a high consumption of olive oil, vegetables, fruits, legumes, and grains; moderate fish and dairy intake; and low meat consumption, could alleviate these symptoms. This study aims to study the prevalence of maternal digestive symptoms in the different pregnancy trimesters and to examine the association between baseline MedDiet adherence and the prevalence of maternal digestive symptoms during pregnancy.

**Methods:**

A secondary analysis of the Walking Preg_Project trial was conducted in a cohort of adult Spanish pregnant women (*N* = 237) who provided data about MedDiet adherence and maternal digestive symptoms (nausea, vomiting, constipation) at baseline (12th ), 19^th,^ and 32nd Gestational Week (GW). MedDiet adherence was appraised through a 13-item questionnaire and categorized into low, medium, and high adherence. Digestive symptoms were assessed by the *Pregnancy Symptoms Inventory*. The association between baseline MedDiet adherence and maternal digestive symptoms was evaluated through adjusted multinomial analysis.

**Results:**

Differences among MedDiet adherence categories were considerable during pregnancy. Some of the greatest decreases were observed in high adherence to MedDiet in comparison with low MedDiet adherence at 32nd GW vs. 19th GW for the prevalence of nausea (10.0% vs. 18.8%, *p* < 0.001) and vomiting (5.0% vs. 8.7%, *p* < 0.001). Constipation remained during pregnancy. There was no significant association between the baseline MedDiet adherence and maternal gastrointestinal symptoms.

**Conclusion for Practice:**

Baseline adherence to the MedDiet was not proven to influence nausea, vomiting, and constipation during pregnancy. For all MedDiet adherence groups, nausea and vomiting prevalence decreased throughout pregnancy, but not constipation.

**Supplementary Information:**

The online version contains supplementary material available at 10.1007/s10995-025-04078-7.

## Significance

Maternal gastrointestinal symptoms affect a large percentage of pregnant women during pregnancy. There are known different pharmaceutical measures against them, but very little is known about non-pharmaceutical ones. The MedDiet offers a broad range of food components that may collaborate to reduce gastrointestinal symptoms based on their physiology. Although the present study has not proven the influence of MedDiet on maternal gastrointestinal symptoms, the study adds a new line of research on this issue.

**Supplementary Information:**

The online version contains supplementary material available at 10.1007/s10995-025-04078-7.

## Background

Physical, mental, and social changes occur during pregnancy, affecting, in many cases, women´s well-being (Lagadec et al., 2018). During pregnancy, digestive symptoms are common, with nausea and vomiting occurring in about 70% of cases (Einarson et al., 2013), while constipation affects between 11% and 38% of pregnant women (Cullen & O’Donoghue, 2007). These symptoms are usually self-limiting but can impair women’s quality of life, affecting their daily activities and their social relationships (Attard et al., 2002) and represent an economic burden for the healthcare system, increasing sick leave and consumption of health resources (Piwko et al., 2013).

The reasons for nausea and vomiting during pregnancy are still not fully understood. High progesterone levels during pregnancy seem to promote the relaxation of the pylorus and decrease the motility of the small bowel, which could cause nausea and vomiting (Gomes et al., 2018). It is also suggested that genetic predisposition and other placentally-mediated mechanisms are involved in the motility process (Bustos et al., 2017). Furthermore, it has been observed that a high level of β-HCG hormone seems to be linked to suffering hyperemesis gravidarum (Mamesah et al., 2019), since multiple pregnancies and molar pregnancies are associated with a higher incidence of hyperemesis gravidarum (Mitsuda et al., 2019). Constipation during pregnancy is also influenced by high levels of progestins, and specifically progesterone, which decreases the motility in the small and large bowels and, along with estrogen, promotes the action of aldosterone, increasing colonic water absorption. Furthermore, as the pregnancy progresses the increased size of the uterus can obstruct defecation and cause constipation (Gomes et al., 2018). Moreover, the adoption of sedentary habits leading to reduced levels of physical activity has been suggested as a significant contributor to constipation during pregnancy (Shi et al., 2015).

To mitigate these symptoms during pregnancy, it is necessary to explore strategies beyond pharmacological interventions. In this sense, diet is a modifiable factor that contributes to gastrointestinal health. In recent years, the Mediterranean diet (MedDiet) has been described as a healthy dietary pattern with a wide range of benefits for human health (Anania et al., 2018; Gantenbein & Kanaka-Gantenbein, 2021; Martínez-González et al., 2019; Sánchez-Villegas et al., 2013; Willett, 2021). Among them, MedDiet influences gut homeostasis by providing a high amount of dietary fiber, prebiotics, and antioxidants, among other essential nutrients (Merra et al., 2021), being considered an optimal diet during pregnancy (Zaragoza-Martí et al., 2022). Guidelines match the importance of establishing a healthy diet pattern during pregnancy as it impacts gestational wellness (Tsakiridis et al., 2020).

A recent study carried out in Italy shows a direct relationship between low adherence to the MedDiet and gastrointestinal symptoms in the general population (Zito et al., 2016). However, although some trials have associated adherence to the MedDiet with numerous benefits during pregnancy, such as protection against gestational diabetes mellitus (De La Torre et al., 2019; Flor-Alemany et al., 2021, 2022; Melero et al., 2020), the effects on the gastrointestinal symptoms observed in the general population have not been yet analyzed in pregnant women.

The present study is aligned with objective 3 “Good health and Well-being” of Sustainable Development Goals (SDG) created by World Health Organization (WHO) (United Nations, 2017) aiming to provide light about the management and possible prevention of maternal digestive symptoms. Besides contribute to know more about developing recommendations by The National Institute for Health and Care Excellence (NICE).

The study aims to: (1) assess the prevalence of maternal gastrointestinal symptoms in the different pregnancy trimesters, and (2) to examine the association between baseline MedDiet adherence and the prevalence of maternal digestive symptoms during pregnancy.

## Methods

### Design of the Study

A secondary analysis of the Walking_Preg Project (WPP) study was performed (Benito-Villena et al., 2022). Initially, WPP was a randomized controlled parallel clinical trial investigating the effect of a walking promotion program on insomnia. More detailed information concerning the intervention can be found in the study protocol (Amezcua-Prieto et al., 2020).

### Participants and Data Collection

**Fig. 1:**
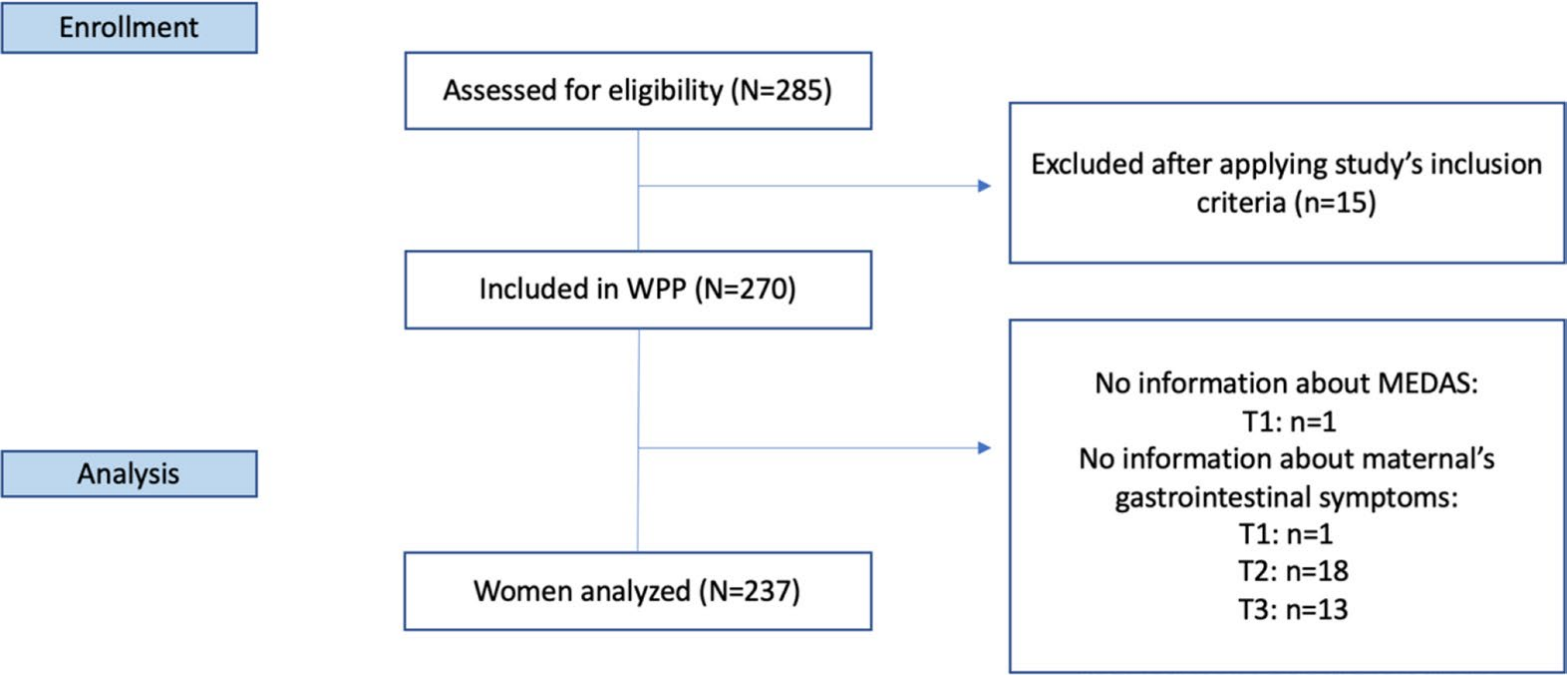
Study flow-chart. Abbreviations: WPP: Walking_Preg_Project trial; Mediterranean diet adherence assessment without wine (MEDAS); T: Interview time; T1: First trimester (baseline); T2: Second trimester; T3: Third trimester

Eligible participants were pregnant women from Granada (Spain). Inclusion criteria were healthy adult pregnant women (18–49 years old) who attended a public third level Maternity Hospital with sedentary habits (< 5 days/week of moderate-vigorous physical activity at least > 30 min.; equivalent to < 7000 steps/day) and without insomnia or taking medicines for sleeping problems. More details about selection criteria can be found in the WPP protocol study (Amezcua-Prieto et al., 2020). The WPP study included 270 randomized pregnant women. For the present analysis, we selected those women who had completed a 13-item questionnaire (*n* = 269) to measure baseline adherence to the MedDiet (12th Gestational Week (GW). Among these women, those who failed (*n* = 32) to complete the Pregnancy Symptoms inventory (PSI) at each trimester of pregnancy (12th, 19^th,^ and 32nd GW) were excluded from this sub-study. Finally, 237 women were analyzed (Fig. 1).

### MedDiet Adherence Assessment

Data on adherence to the MedDiet was assessed by the adapted Mediterranean Dietary Adherence Screener (MEDAS) at the 12th GW, which captured dietary habits from the month prior to pregnancy to baseline (12th GW). MEDAS was also completed at 19th and 32nd GW. MEDAS is a 14-item questionnaire validated for the Spanish adult population (Schröder et al., 2011) and previously used in pregnant women as a 13-item questionnaire, excluding wine consumption, with a minimum score of 0 and a maximum of 13. Wine consumption was excluded from the MedDiet adherence assessment due to recommendations by organizations such as the American College of Obstetricians and Gynecologists (American College of Obstetricians and Gynecologists, 2011), which advises against alcohol intake during pregnancy. Other authors have employed the same assessment tool (Gesteiro et al., 2012).

MEDAS items scored as 1 point are the use of ≥ 4 tablespoons/day of extra virgin olive oil, fruits ≥ 3 servings/day, vegetables ≥ 2 servings/day (at least one raw), fish ≥ 3 servings/week, legumes ≥ 3 servings/week, nuts ≥ 3 servings/week, white meat preference, Sauté “sofrito” ≥ 3 servings/week, butter or margarine < 1 serving/day, red meat < 1 serving/day, sugary carbonated drinks < 1 serving/day, and industrial pastries < 2 servings/week. 0 points are recorded for each item if the conditions are not met.

The adherence to MedDiet was classified into three categories: low (0–7 points), moderate (8–10 points), and high (11–13 points) adherence, based on previous studies where ≤ 7 points were considered a low MedDiet adherence (Benítez Benítez et al., 2016; Guillén Alcolea et al., 2021). Participants were distributed into each MedDiet adherence group according to their score: *n* = 69 in the low MedDiet adherence group, *n* = 127 in the moderate group, and *n* = 40 in the high MedDiet adherence group.

### Outcome Variables

Data about nausea, vomiting, and constipation were collected by trained staff using the Pregnancy Symptom Inventory (PSI) (Foxcroft et al., 2013; Oviedo-Caro et al., 2017) at the 12th, 19th, and 32nd GW. The PSI is a 41-item scale that registers the frequency of symptoms that appear in each trimester of pregnancy and their daily impact (not considered in the present study). The frequency of symptoms is measured by a Likert scale from 0 to 3: “never”, “rarely”, “sometimes” and “often”. The PSI maximum score is 123 and it represents a large range of symptoms.

Considering the Likert scale of PSI, the maternal digestive symptoms were recategorized into two categories: presence of symptoms (including “sometimes” and “often” and absence of symptoms (including “never” and “rarely”).

### Covariate Assessment

Trained WPP trial staff compiled information on sociodemographic and lifestyle variables at the first interview (12th GW). Collected variables were age, Body Mass Index (BMI) (kg/m^2^), number of previous children (parity = 0 /1/ ≥2), educational level (primary / high school / university), stable couple (no / yes and not cohabiting / yes and cohabiting), current smoking habit (yes / no), and social class (calculated considering the occupation of women and their partner and classified into one of the five classes established by the Spanish Society of Epidemiology) (Álvarez-Dardet, 1995). Besides, physical activity level was assessed through the short International Physical Activity Questionnaire (IPAQ) (Sanda et al., 2017) classifying the participants into three categories according to their activity per week (light / moderate / vigorous).

### Data Analysis

All the statistical analyses were performed using the STATA software (version 15.1, StataCorp, United States of America, http://www.stata.com), and p-values < 0.05 were considered statistically significant. We used the WPP database generated in February 2022 for the current analysis. Data are presented as mean ± standard deviation (SD) for continuous variables or number and percentage for categorical variables. To describe the characteristics of participants, Shapiro-Wilk and Kruskall-Wallis tests were performed for continuous variables and a Chi-squared test for categorical variables. Chi-squared test was also performed to analyze the prevalence (P) of maternal gastrointestinal symptoms in each trimester (12th, 19^th,^ and 32nd GW) according to MedDiet adherence. The incidence of new gastrointestinal symptoms and persistence across trimesters was analyzed to determine trends within and between MedDiet adherence categories. Incidence was defined as the appearance of a new symptom in participants who were symptom-free in the previous trimester. Persistence was categorized as the continuation of symptoms reported across consecutive trimesters. These analyses were conducted for nausea, vomiting, and constipation across three-time points: the 12th, 19th, and 32nd GW. The results were stratified by baseline MedDiet adherence categories, and statistical significance was determined using the Chi-squared test. To see changes within MedDiet adherence groups in 12th vs. 19th and 12th vs. 32nd GW for each gastrointestinal maternal symptom, we used the McNemar test.

The association between MedDiet adherence categories and maternal digestive symptoms was evaluated through binomial and multinomial logistic regression models, taking Low MedDiet adherence as the reference category. Crude and adjusted Relative Risk (RR)/Odds Ratio (OR), and 95% Confidence Intervals (CI) were calculated. Regression models were adjusted for potential confounders based on prior knowledge (Álvarez-Fernández et al., 2021; Ioannidou et al., 2019; Lacasse et al., 2008; Owe et al., 2019; Rungsiprakarn et al., 2014): maternal age, pre-pregnancy BMI, smoking habit, physical activity level, social class, parity, allocation group, civil status and educational level.

## Results

### Characteristics of the Study Subjects

Participants’ baseline characteristics are described in Table 1. Compared to pregnant women with moderate and high MedDiet adherence, the group of low MedDiet adherence was younger (*p* = 0.003), had a higher BMI (*p* = 0.018), smoked more at the beginning of pregnancy (*p* < 0.001), did not live in a stable couple (*p* = 0.003), had a lower educational level (*p* < 0.001) and a lower socioeconomic class (*p* < 0.001). In contrast, in the high MedDiet adherence group, more women had university studies (*p* < 0.001) and belong to the high (I) social class (*p* < 0.001) compared to low MedDiet adherence. No differences were found in physical activity levels among groups.

**Table 1:** Study participant’s characteristics according to baseline categories of MedDiet adherence

		Overall sample (*N* = 237)	Low (*n* = 69)	Moderate (*n* = 128)	High (*n* = 40)	*p*-value
Age, mean (SD)		32.03 (5.2)	30.23 (4.9)	32.59 (5.4)	33.33 (3.9)	0.003*
Pre-pregnancy BMI (kg/m^2^), mean (SD)		26.19 (5.7)	27.92 (6.8)	25.73 (5.1)	24.69 (4.6)	0.018*
Parity, n (%)	0 children	125 (52.7)	42 (60.9)	58 (45.3)	25 (62.5)	0.002*
	1 child	79 (33.3)	12 (17.4)	56 (43.8)	11 (27.5)	
	≥ 2 children	33 (14.0)	15 (21.7)	14 (10.9)	4 (10.0)	
Stable couple, n (%)	No	8 (3.4)	6 (8.7)	2 (1.6)	0 (0.0)	0.003*
	Yes (non-cohabit)	5 (2.1)	4 (5.8)	1 (0.8)	0 (0.0)	
	Yes (cohabit)	224 (94.5)	59 (85.5)	125 (97.7)	40 (100.0)	
Educational level, n (%)	Analphabet	0 (0.0)	0 (0.0)	0 (0.0)	0 (0.0)	< 0.001*
	Primary	53 (22.4)	26 (37.7)	23 (18.0)	4 (10.0)	
	High school	86 (36.3)	26 (37.7)	49 (38.3)	11 (27.5)	
	University	98 (41.3)	17 (24.6)	56 (43.8)	25 (62.5)	
Social class, n (%)	I	39 (16.5)	4 (5.8)	23 (18.0)	12 (30.0)	< 0.001*
	II	36 (15.2)	10 (14.5)	18 (14.1)	8 (20.0)	
	III	47 (19.8)	10 (14.5)	29 (22.7)	8 (20.0)	
	IV	84 (35.4)	29 (42.0)	50 (39.1)	5 (12.5)	
	V	31 (13.1)	16 (23.2)	8 (6.2)	7 (17.5)	
Actual smoke, n (%)	Yes	26 (11.0)	17 (24.6)	9 (7.0)	0 (0.0)	< 0.001*
Physical activity, n (%)	Light	67 (28.3)	25 (36.2)	35 (27.3)	7 (17.5)	*0.251*
	Moderate	136 (57.4)	37 (53.6)	72 (56.2)	27 (67.5)	
	Vigorous	34 (14.3)	7 (10.1)	21 (16.4)	6 (15.0)	

*Abbreviations* S.D.: Standard Deviation; BMI: Body Mass Index. Low: Low MedDiet adherence = 0–7 points; Moderate: Moderate MedDiet adherence = 8–10 points; High: High MedDiet adherence = 11–13 points; p-value: Pearson chi-square and Kruskall-Wallis tests were performed to evaluate differences in categorical and continuous variables, respectively. Values presented in bald showed a statistically significant association (*p* < 0.05)

### Prevalence of Maternal Digestive Symptoms According To Baseline MedDiet Adherence Categories

The prevalence of maternal digestive symptoms according to MedDiet adherence is shown in Table 2. Prevalence of nausea and vomiting significantly decreased during pregnancy within MedDiet adherence categories (< 0.005). Differences in the prevalence of nausea and vomiting throughout pregnancy were significant among MedDiet adherence categories. Some of the greatest decreases were observed in high adherence to MedDiet in comparison with low MedDiet adherence at 32nd GW vs. 19th GW for the prevalence of nausea (10.0% vs. 18.8%, *p* < 0.001) and vomiting (5.0% vs. 8.7%, *p* < 0.001).

**Table 2:** Prevalence, incidence and persistence digestive symptoms across pregnancy according to baseline MedDiet adherence

		Overall sample (*N* = 237)	Low (*n* = 69)	Moderate (*n* = 127)	High (*n* = 40)	*p*-value^1^
**Nausea**						
12th GW, n (%)		133 (56.1)	34 (49.3)	76 (59.4)	23 (57.5)	*0.388*
19th GW, n (%) Incident cases 12th − 19th, n (%)		56 (23.6) 6 (10.7)	18 (26.1) 3 (16.7)	32 (25.0) 3 (9.4)	6 (15.0) 0 (0.0)	*0.365* *0.403*
32nd GW, n (%) Incident cases 19th – 32th, n (%) Persistence 12th − 32nd GW, n (%)		41 (17.3) 6 (14.6) 22 (9.3)	13 (18.8) 2 (15.4) 8 (11.6)	24 (18.8) 4 (16.7) 10 (7.9)	4 (10.0) 0 (0.0) 4 (10.0)	*0.408* *0.734* *0.654*
p-value^2^	12th vs. 19th GW		< 0.001*	< 0.001*	< 0.001*	
	12th vs. 32nd GW		< 0.001*	< 0.001*	< 0.001*	
**Vomiting**						
12th GW, n (%)		72 (30.4)	23 (33.3)	37 (28.9)	12 (30.0)	*0.811*
19th GW, n (%) Incident cases 12th − 19th, n (%**)		34 (14.3) 12 (35.3)	10 (14.5) 3 (30.0)	20 (15.6) 8 (40.0)	4 (10.0) 1 (25.0)	*0.675* *0.783*
32nd GW, n (%) Incident cases 19th – 32th, n (%**) Persistence until 32nd GW, n (%)		20 (8.4) 3 (15.0) 10 (4.2)	6 (8.7) 2 (33.3) 2 (2.9)	12 (9.4) 1 (8.3) 6 (4.7)	2 (5.0) 0 (0.0) 2 (5.0)	*0.676* *0.289* *0.826*
p-value^2^	12th vs. 19th GW		< 0.001*	< 0.001*	0.004*	
	12th vs. 32nd GW		< 0.001*	< 0.001*	< 0.001*	
**Constipation**						
12th GW, n (%)		101 (42.6)	33(47.8)	52 (40.6)	16 (40.0)	*0.581*
19th GW, n (%) Incident cases 12th − 19th, n (%**)		91 (38.4) 30 (33.0)	28 (40.6) 13 (46.4)	51 (39.8) 15 (29.4)	12 (30.0) 2 (16.7)	*0.486* *0.120*
32nd GW, n (%) Incident cases 19th – 32th, n (%**) Persistence until 32nd GW, n (%)		86 (36.3) 24 (27.9) 37 (15.6)	28 (40.6) 5 (17.9) 9 (13.0)	48 (37.5) 17 (35.4) 23 (18.1)	10 (25.0) 2 (20.0) 5 (12.5)	*0.242* *0.247* *0.611*
p-value^2^	12th vs. 19th GW		*0.131*	*0.862*	*0.706*	
	12th vs. 32nd GW		*0.433*	*0.286*	*0.593*	

*Abbreviations* SD: standard deviation; GW: gestational week.; Low: Low MedDiet adherence = 0–7 points; Moderate: Moderate MedDiet adherence = 8–10 points; High: High MedDiet adherence = 11–13 points; p-value^1^: Pearson chi-square test comparison between categories; p-value^2^: Prevalence McNemar test comparison within categories. Values presented in bald showed a statistically significant association (*p* < 0.05)

The prevalence of constipation remained constant throughout pregnancy in all categories of MedDiet adherence (*p* > 0.05). Notably, constipation prevalence in the low adherence group was the same at 12th (*P* = 40.6%) and 19th (*P* = 40.6%), whereas constipation prevalence in the high MedDiet adherence group was 30.0% and 25.9%, respectively.

The incidence and persistence of gastrointestinal symptoms were analyzed (Supplementary Table 1). No significant differences were observed in the incidence and persistence of symptoms across MedDiet adherence categories.

### Association Between Maternal Digestive Symptoms Throughout Pregnancy and Baseline Categories of Meddiet Adherence

Table 3 shows no association between MedDiet adherence and maternal digestive symptoms throughout pregnancy. Pregnant women from moderate and high MedDiet adherence seemed to have more nausea at 12th GW (Moderate MedDiet RRa = 1.98 CI95% 0.98-4.00; High MedDiet RRa = 2.11 95% CI 0.84–5.29) and less nausea at 32nd GW in the high MedDiet (RRa = 0.58 95% CI 0.15–2.15), compared with the low adherence group. Women with a higher MedDiet adherence show minor constipation at 32nd GW (RRa = 0.65 95% CI 0.25–1.69). However, the results were not statistically significant. In this study, MedDiet adherence was not associated with maternal digestive symptoms throughout pregnancy.

**Table 3:** Association between maternal digestive symptoms throughout pregnancy and categories of MedDiet adherence *Abbreviations* GW: gestational week; ORc: crude odds ratio; ORa: adjusted odds ratio; IC: interval confidence; ref: reference category. Low adherence = 0–7 points; moderate adherence = 8–10 points; high adherence = 11–13 points. Logistic regression models were performed to get adjusted OR and their 95% IC. ORa were adjusted by educational level, stable couple, parity, social class, smoke, IPAQ, intervention group, age and BMI

Multinomial Logistic regression models		Low (*n* = 69)		Moderate (*n* = 127)		High (*n* = 40)	
		ORc	ORa	ORc	ORa	ORc	ORa
**Nausea**	12th GW	1 (ref)	1 (ref)	0.66 (0.37–1.20)	0.52 (0.25–1.05)	0.72 (0.32–1.57)	0.47 (0.18–1.20)
	19th GW	1 (ref)	1 (ref)	0.94 (0.49–1.87)	0.78 (0.34–1.82)	0.50 (0.17–1.33)	0.48 (0.13–1.59)
	32nd GW	1 (ref)	1 (ref)	0.99 (0.48–2.15)	0.81 (0.32–2.10)	0.48 (0.13–1.47)	0.56 (0.12–2.20)
**Vomiting**	12th GW	1 (ref)	1 (ref)	0.81 (0.44–1.54)	1.07 (0.50–2.37)	0.86 (0.36–1.97)	1.33 (0.48–3.69)
	19th GW	1 (ref)	1 (ref)	1.09 (0.49–2.58)	0.98 (0.37–2.74)	0.66 (0.17–2.12)	0.75 (0.16–3.08)
	32nd GW	1 (ref)	1 (ref)	1.10 (0.41–3.28)	1.77 (0.48–7.72)	0.55 (0.08–2.54)	2.04 (0.19–18.2)
**Constipation**	12th GW	1 (ref)	1 (ref)	0.75 (0.41–1.35)	1.09 (0.54–2.23)	0.73 (0.33–1.59)	1.06 (0.42–2.67)
	19th GW	1 (ref)	1 (ref)	0.97 (0.54–1.77)	1.25 (0.61–2.57)	0.63 (0.27–1.42)	0.83 (0.31–2.18)
	32nd GW	1 (ref)	1 (ref)	0.88 (0.48–1.61)	0.87 (0.42–1.83)	0.49 (0.20–1.13)	0.54 (0.19–1.45)

### Association between Maternal Digestive Symptoms Throughout Pregnancy: Low Versus Moderate-High Baseline MedDiet Adherence

Table 4 was constructed to analyze whether baseline adherence to MedDiet (low versus moderate-high) had an impact on maternal gastrointestinal symptoms, rather than a MedDiet classification in three categories (low, middle, and high). No great changes were observed compared to Table 3. Pregnant women from moderate-high MedDiet adherence still seemed to have more vomiting at the 12th GW (ORa = 1.05 95% CI 0.51–2.22) but less nausea at the 32nd GW (ORa = 0.75 95% CI 0.31–1.89); and minor constipation at 32nd GW (ORa = 0.78 95% CI 0.38–1.61).

**Table 4:** Association between maternal digestive symptoms throughout pregnancy and low versus moderate-high MedDiet adherence

Logistic regression models		Low (*n* = 69)		Moderate-High (*n* = 167)	
		ORc	ORa	ORc	ORa
**Nausea**	12th GW	1 (ref)	1 (ref)	0.68 (0.38–1.19)	0.54 (0.27–1.07)
	19th GW	1 (ref)	1 (ref)	0.88 (0.44–1.61)	0.71 (0.32–1.63)
	32nd GW	1 (ref)	1 (ref)	0.86 (0.42–1.83)	0.75 (0.31–1.89)
**Vomiting**	12th GW	1 (ref)	1 (ref)	0.82 (0.45–1.52)	1.05 (0.51–2.22)
	19th GW	1 (ref)	1 (ref)	0.98 (0.45–2.27)	0.93 (0.36–2.55)
	32nd GW	1 (ref)	1 (ref)	0.96 (0.37–2.82)	1.68 (0.47–7.20)
**Constipation**	12th GW	1 (ref)	1 (ref)	0.74 (0.42–1.31)	1.09 (0.56–2.17)
	19th GW	1 (ref)	1 (ref)	0.88 (0.50–1.57)	1.13 (0.57–2.29)
	32nd GW	1 (ref)	1 (ref)	0.77 (0.43–1.38)	0.78 (0.38–1.61)

*Abbreviations* GW: gestational week; ORc: crude Odds Ratio; ORa: adjusted Odds Ratio; IC: Interval Confidence; Abbreviations: **ref**: reference category; **Low**: Low adherence = 0–7 points; **Moderate-High**: Moderate-High adherence = 8–13 points. ORa: adjusted by educational level, stable couple, parity, social class, smoke, IPAQ, intervention group, age and BMI

## Discussion

In the present study, the prevalence of maternal gastrointestinal symptoms was similar between MedDiet adherence groups. The prevalence of nausea and vomiting decreased with trimesters, with no significant differences in constipation. A smaller decrease in constipation prevalence was observed in all adherence groups. There was no association between MedDiet adherence and the frequency of maternal gastrointestinal symptoms in any trimester of pregnancy. Although we calculated the incidence and persistence of these symptoms across MedDiet adherence categories, no significant differences were observed. Our findings highlight that while gastrointestinal symptoms are prevalent during pregnancy, adherence to the MedDiet does not significantly influence their onset or continuation.

Few studies have found an association between diet and maternal digestive symptoms. A cross-sectional study showed a positive association between animal calorie intake, animal protein, animal fat, animal carbohydrates, sugars and sweeteners, and total fat with nausea and vomiting rates during pregnancy. In contrast, cereals and pulses were negatively associated with these symptoms (Pepper & Roberts, 2006).

Fiber could be an important element in preventing constipation during pregnancy as different studies in pregnant women showed that an adequate fiber intake prevents constipation (Reijonen et al., 2022a; Trottier et al., 2012), although without an association with nausea and vomiting (Reijonen et al., 2022). Likewise, fiber and prebiotics also protect against constipation in the general population (Katsirma et al., 2021; Slavin, 2013). Following that, a cohort study of pregnant women (*n* = 804) highlighted a tolerance of fiber with nausea and vomiting, and without resulting in any exacerbation (Pretorius & Palmer, 2020). This study also shows that pregnant women who had a higher fiber intake obtained this through fruits and vegetables.

The MedDiet is characterized by high consumption of vegetable portions that include fruit, vegetables, pulses, whole grains, nuts, and olive oil, providing an amount of fiber, prebiotics, and vegetable fat, among other nutrients (Davis et al., 2015). Thus, the MedDiet contains the associations of the food components shown by the previous studies (Katsirma et al., 2021; Pepper & Roberts, 2006; Pretorius & Palmer, 2020; Reijonen et al., 2022a; Reijonen et al., 2022; Slavin, 2013; Trottier et al., 2012) to be related to gastrointestinal symptoms and therefore could be protective against constipation, nausea, and vomiting with no exacerbation. However, we could not find any association between adherence to MedDiet and those symptoms.

Differences in sociodemographic characteristics between MedDiet adherence groups were also observed in a prospective series of 1175 pregnant women, where high MedDiet adherence was associated with greater age, higher social class, and higher educational level (Olmedo-Requena et al., 2014). These associations with MedDiet adherence degrees have also been found in the general population (Hu et al., 2013; Mendonça et al., 2022; Pribisalić et al., 2021).

Gastrointestinal changes during pregnancy are described as a natural process of muscle relaxation and gastric compression, which leads to gastrointestinal symptomatology such as nausea, and vomiting, usually lasting until the 20th GW (Carrillo-Mora et al., 2021), and constipation, more prevalent among 12-36th GW (Shin et al., 2015). This matches with the higher overall prevalence of both nausea and vomiting at the first and second trimesters, and the constant prevalence of constipation observed throughout pregnancy in the WPP study, which indicates a physiological basis in the results.

Our study is a secondary analysis of the WPP study, which was performed during the COVID-19 pandemic. This fact must be considered a possible limitation since the pandemic altered people’s lifestyles and dietary habits, reporting a poor nutritional status as both obesity and underweight/unintended weight (Antwi et al., 2021). Considering that the exposition of the present study is the initial adherence to MedDiet (12th ), the COVID-19 pandemic was an unusual situation that could directly modify the adherence to MedDiet. This way, the pandemic situation could also have led to information bias where Mediterranean diet markers such as hydroxytyrosol of olive oil or omega-6/omega-3 balance could have provided stronger information about MedDiet adherence (Ly et al., 2021; Martinotti et al., 2021; Urquiaga et al., 2017). In our study, adherence to the MedDiet improved during pregnancy, increasing from 8.22 points at the 12th GW to 9.10 and 9.22 points at the 19th and 32nd GW, respectively (data not shown). All participants were recruited and followed during the pandemic, and the conditions for adherence change were consistent across the cohort. The observed improvement in MedDiet adherence scores over the course of pregnancy reflects a positive trend toward healthier eating behaviors during this period instead of the COVID-19 pandemic. This increase may be attributed to increased maternal awareness of dietary recommendations. Nevertheless, no significant associations were found between adherence to the MedDiet and the prevalence of gastrointestinal symptoms.

The present study is the first looking for an association between MedDiet adherence and maternal gastrointestinal symptoms. Although no conclusive results have been observed, we found a practical implication for counseling; low educational level and social class presented less adherence to the MedDiet, which may lead to both child and mother health complications (Biagi et al., 2019; De La Torre et al., 2019; Zaragoza-Martí et al., 2022).

In the investigated cohort of pregnant women, the prevalence of nausea, vomiting, and constipation was not influenced by MedDiet adherence. Nausea and vomiting prevalence decreased within trimesters in all groups and was not conclusive for constipation. The MedDiet does not appear to be associated with the frequency of the maternal digestive symptoms investigated. However, future research should explore the preventive role of MedDiet on gastrointestinal symptom incidence and its therapeutic potential, using prospective designs with clear temporality and standardized measures.

## Electronic Supplementary Material

Below is the link to the electronic supplementary material.

Supplementary Material 1

## Data Availability

The raw data and materials can be obtained on request from the corresponding author.

## Abbreviations

BMI: Body Mass Index
CI: Confidence Interval
GW: Gestational Week
IPAQ: International Physical Activity Questionnaire
MEDAS: Mediterranean Diet Adherence Screener
MedDiet: Mediterranean Diet
OR: Odds Ratio
ORc: Crude Odds Ratio
ORa: Adjusted Odds Ratio
PSI: Pregnancy Symptoms inventory
RCT: Randomized Controlled Trial
SD: Standard Deviation
WPP: Walking Preg_Project
