# Supplementary material for: Impact of Mediterranean Diet Adherence in Early Pregnancy on Nausea, Vomiting, and Constipation
Source: Matern Child Health J. 2025 Apr 7;29(5):639–49. doi: 10.1007/s10995-025-04078-7 (PMC12098518; doi:10.1007/s10995-025-04078-7)
Supplement: Supplementary file 1 — Supplementary Material 1 [file 10995_2025_4078_MOESM1_ESM.docx]

**Supplementary Table 1. Incidence and persistence of digestive symptoms across pregnancy according to baseline MedDiet adherence.**

|  | Overall sample  (N=237) | Low  (n=69) | Moderate  (n=127) | High  (n=40) | p-value^1^ |
| --- | --- | --- | --- | --- | --- |
| **Incidence** |  |  |  |  |  |
| **Nausea** |  |  |  |  |  |
| Incident cases 12^th^ - 19^th^, n (%) ^a^ | 6 (10.7) | 3 (16.7) | 3 (9.4) | 0 (0.0) | *0.403* |
| Incident cases 19^th^- 32^nd^, n (%) ^b^ | 6 (14.6) | 2 (15.4) | 4 (16.7) | 0 (0.0) | *0.734* |
| **Vomiting** |  |  |  |  |  |
| Incident cases 12^th^ - 19^th^, n (%) | 12 (35.3) | 3 (30.0) | 8 (40.0) | 1 (25.0) | *0.783* |
| Incident cases 19^th^- 32^nd^, n (%) | 3 (15.0) | 2 (33.3) | 1 (8.3) | 0 (0.0) | *0.289* |
| **Constipation** |  |  |  |  |  |
| Incident cases 12^th^ - 19^th^, n (%) | 30 (33.0) | 13 (46.4) | 15 (29.4) | 2 (16.7) | *0.120* |
| Incident cases 19^th^- 32^nd^, n (%) | 24 (27.9) | 5 (17.9) | 17 (35.4) | 2 (20.0) | *0.247* |
| **Persistence** |  |  |  |  |  |
| **Nausea** |  |  |  |  |  |
| Persistence 12^th^ - 32^nd^, n (%) ^c^ | 22 (9.3) | 8 (11.6) | 10 (7.9) | 0 (0.0) | *0.734* |
| **Vomiting** |  |  |  |  |  |
| Persistence 12^th^ - 32^nd^, n (%) | 10 (4.2) | 2 (2.9) | 6 (4.7) | 2 (5.0) | *0.826* |
| **Constipation** |  |  |  |  |  |
| Persistence 12^th^ - 32^nd^, n (%) | 37 (15.6) | 9 (13.0) | 23 (18.1) | 5 (12.5) | *0.611* |

Abbreviations: Low: Low MedDiet adherence=0-7 points; Moderate: Moderate MedDiet adherence=8-10 points; High: High MedDiet adherence=11-13 points; p-value^1^: Pearson chi-square test comparison between categories; ^a^ New cases at 19^th^, not present at 12^th^ gestational week; ^b^ New cases at 32^nd^, not present at 19^th^ gestational week; ^c^ Persistent gastrointestinal symptoms cases, present at 12 ^th^, 19 ^th^ and 32 ^nd^ gestational week.
